# Approximating Attractors of Boolean Networks by Iterative CTL Model Checking

**DOI:** 10.3389/fbioe.2015.00130

**Published:** 2015-09-08

**Authors:** Hannes Klarner, Heike Siebert

**Affiliations:** ^1^Fachbereich Mathematik und Informatik, Freie Universität Berlin, Berlin, Germany

**Keywords:** Boolean networks, asynchronous dynamics, attractors, CTL model checking, ASP, signaling, gene regulation

## Abstract

This paper introduces the notion of approximating asynchronous attractors of Boolean networks by minimal trap spaces. We define three criteria for determining the quality of an approximation: “faithfulness” which requires that the oscillating variables of all attractors in a trap space correspond to their dimensions, “univocality” which requires that there is a unique attractor in each trap space, and “completeness” which requires that there are no attractors outside of a given set of trap spaces. Each is a reachability property for which we give equivalent model checking queries. Whereas faithfulness and univocality can be decided by model checking the corresponding subnetworks, the naive query for completeness must be evaluated on the full state space. Our main result is an alternative approach which is based on the iterative refinement of an initially poor approximation. The algorithm detects so-called autonomous sets in the interaction graph, variables that contain all their regulators, and considers their intersection and extension in order to perform model checking on the smallest possible state spaces. A benchmark, in which we apply the algorithm to 18 published Boolean networks, is given. In each case, the minimal trap spaces are faithful, univocal, and complete, which suggests that they are in general good approximations for the asymptotics of Boolean networks.

## 1 Introduction

Boolean and multi-valued networks are frequently used to model the dynamics of biological processes that involve gene regulation and signal transduction. The dynamics of such models is captured by the state transition graph, a directed graph that relates states to potential successor states. Different transition relations have been suggested, among them the synchronous update of Kauffman (1993) and the asynchronous update of Thomas (1991). An important type of prediction that can be obtained from such models concerns the long-term behavior of the represented processes. Formally, the long-term behaviors correspond to the minimal trap sets of the state transition graph which are also called its attractors.

Recently, we have suggested to compute the minimal trap spaces of a network to obtain an approximation for its cyclic attractors (Klarner et al., 2014) and proposed an efficient, *Answer Set Programing* (ASP)-based method for their computation. This paper presents an iterative algorithm that combines *Computation Tree Logic* (CTL) model checking with the computation of minimal trap spaces to determine the quality of the approximation.

The paper is organized as follows. Section 2 recapitulates the background including directed graphs, the dynamics of Boolean networks, trap spaces, and model checking. It is only meant to introduce the notation required for the subsequent sections. Section 3 briefly discusses the attractor detection problem. In Section 4, we describe three conditions under which there is a one-to-one correspondence between the minimal trap spaces and the attractors of a network, and how CTL queries may be used to decide whether they hold. The computationally most challenging one is treated in Section 5. In Section 6, we present a full analysis of a MAPK signaling network as well as the results for 18 Boolean models that are currently in the ginsim repository. Section 7 is an outlook and conclusion. There is a Supplementary Material that contains proofs for the formal statements in the main text.

## 2 Background

### 2.1 Directed graphs

Since several aspects of Boolean networks involve directed graphs (digraphs) we introduce the general terminology. Let (*V*, *A*) be a digraph with vertices *V* and arcs *A* ⊆ *V* × *V*.

An **infinite path** in (*V*, *A*) is an infinite sequence of vertices π = (*v*_0_, *v*_1_, …) such that (*v_i_*, *v_i_*_+1_) ∈ *A* for all *i* ∈ ℕ_0_. **Finite paths** are defined analogously for finite sequences. In particular, π = (*v*_0_) is an admissible finite path. We denote the set of all infinite paths that start in *v* ∈ *V* by *InfPaths*(*v*) and finite paths by *FinPaths*(*v*). The *i*th vertex of π is denoted by π[*i*] : = *v_i_*. For finite paths we denote by *FinPaths*(*u*, *v*) all finite paths that start with *u* and end with *v*. The number of vertices in a finite path π = (*v*_0_, *v*_1_, …, *v_k_*) is denoted by *len*(π) : = *k* + 1.

A vertex *v* ∈ *V* is **reachable** from *u* ∈ *V* iff *FinPaths*(*u*, *v*) ≠ ∅. We denote by *Above*(*v*) the vertices that can reach *v*. A subset *U* ⊆ *V* is **strongly connected** if every *u* ∈ *U* is reachable from every other *v* ∈ *U*. A **strongly connected component** (SCC) is an inclusion-wise maximal subset *U* ⊆ *V* that is strongly connected. We denote the set of SCCs of a digraph by *SCCs* (*V*, *A*). Note that since π = (*v*_0_) is an admissible finite path, every vertex is trivially reachable from itself. Hence, each node belongs to some SCC and *SCCs* (*V*, *A*) is a partition of *V*.

### 2.2 Boolean networks

We consider variables from the Boolean domain B = {0,1}, where 1 and 0 represent the truth values *true* and *false*. A Boolean **expression** *f* over the variables *V* = {*v*_1_, …,*v_n_*} is defined by a formula over the grammar

$$f::=0|1|v|\bar{f}|f_{1}\cdot f_{2}|f_{1}+f_{2}$$

where *v* ∈ *V* signifies a variable, $\bar{f}$ the negation, *f*_1_ ⋅ *f*_2_ the conjunction and *f*_1_ + *f*_2_ the (inclusive) disjunction of the expressions *f*,*f*_1_, and *f*_2_. Given an **assignment** *x* : *V*  → B, an expression *f* can be evaluated to a value *f* (*x*) ∈ B by substituting the values *x*(*v*) for the variables *v* ∈ *V*. If *f* (*x*) = *f* (*y*) for all assignments *x, y* : *V*  → B, we say *f* is **constant** and write *f* = *c*, with *c* ∈ B being the constant value. A **Boolean network** (*V*, *F*) consists of *n* variables *V*  = {*v*_1_, … ,*v_n_*} and *n* corresponding Boolean expressions *F* = { *f*_1_, … , *f_n_*} over *V*. In this context, an assignment *x* : *V* → B is also called a **state** of the network and the **state space** *S* = *S_V_* consists of all possible 2*^n^* states. We specify states by a sequence of *n* values that correspond to the variables in the order given in *V*, i.e., *x* = 110 should be read as *x*(*v*_1_) = 1, *x*(*v*_2_) = 1, and *x*(*v*_3_) = 0. The expressions *F* can be thought of as a function *F* : *S* → *S* governing the network behavior. The **image** *F*(*x*) of a state *x* under *F* is defined to be the state *y* that satisfies *y*(*v_i_*) = *f_i_*(*x*).

The **interaction graph** of a network (*V*, *F*) captures the dependencies between the variables and their expressions. It is a digraph (*V*, →) where → ⊆ *V* × *V* and (*u*, *v*) ∈ → iff there are *x*, *y* ∈ *S* such that *x*(*w*) = *y*(*w*) for all *w* ∈ *V*  ∖ {*u*} and *f_v_*(*x*) ≠ *f_v_*(*y*). As for state transitions we write *u* → *v* iff (*u*,*v*) ∈ →.

The **state transition graph** (STG) of a Boolean network (*V*, *F*) is the digraph (S, →) where the transitions → ⊆ *S* × *S* are obtained from *F* via a given update rule. We usually write *x* → *y* iff (*x*, *y*) ∈ →. We mention two update rules here, the **synchronous rule** and its transition relation ↠ ⊆ *S* × *S*, and the **asynchronous rule** and its transition relation ↪ ⊆ *S* × *S*. The former is defined by *x* ↠ *y* iff *F*(*x*) = *y*. To define ↪ we need the Hamming distance Δ : *S* × *S* → {1, … , *n*} between states which is given by Δ(*x*, *y*) : = |{*v* ∈*V*  | *x*(*v*) ≠ *y*(*v*)}|. We define *x* ↪* y* iff either *x* = *y* and *F*(*x*) = *x* or Δ(*x*,*y*) = 1 and Δ (*y*, *F*(*x*)) < Δ (*x*, *F*(*x*)). In the context of the STG, the expressions *f*  ∈ *F* are also called **update functions**.

A non-empty set *T* ⊆ *S* is a **trap set** of (*S*, →) iff for every *x* ∈ *T* and *y* ∈ *S* with *x* → *y* it holds that *y* ∈ *T*. An inclusion-wise minimal trap set is also called an **attractor** of (S, →). Every trap set contains at least one minimal trap set and therefore at least one attractor. A variable *v* ∈ *V* is **steady** in an attractor *A* ⊆* S* iff *x*(*v*) = *y*(*v*) for all *x*, *y* ∈ *A* and **oscillating** otherwise. We distinguish two types of attractors depending on their size. If *A* ⊆ *S* is an attractor and |*A*| = 1, then *A* is called a **steady state** and if |*A*| > 1, we call it a **cyclic attractor**. The cyclic attractors of (*S*, ↠) are, in general, different from the cyclic attractors of (*S*, ↪). The steady states, however, are identical in both transition graphs because *x* ∈ *S* is steady iff *x* → *x* which is characterized, for both update rules, by the equation *F*(*x*) = *x*. Hence, we may omit the update rule and denote the set of steady states by *S_F_*.

A **subspace** of *S* is characterized by its fixed and free variables. It may be specified by an assignment *p* : *D* → B where *D* ⊆ *V* is the subset of **fixed** variables, *p*(*u*) the value of *u* ∈ *D* and the remaining variables, *V*  ∖ *D*, are said to be **free**. Subspaces are sometimes referred to as “symbolic states” (Siebert, 2011) or “partial states” (Irons, 2006). We specify subspaces like states but allow in addition the symbol ^⋆^ to indicate that a variable is free, i.e., *p* = ^⋆⋆^10 means *D* = {*v*_3_, *v*_4_} and *p*(*v*_3_) = 1, *p*(*v*_4_) = 0. The set *S*^⋆^ = $S_{F}^{⋆}$ denotes all possible 3*^n^* subspaces. States are therefore a special kind of subspace and *S* ⊂ *S*^⋆^ holds. We denote the fixed variables *D* of a specific *p* ∈ *S*^⋆^ by *D_p_*. A subspace *p* references the states *S*[*p*] : = {*x* ∈ *S* | ∀*v* ∈ *D_p_* : *x*(*v*) = *p*(*v*)}. We denote the unique subspace that does not fix any variables by ε ∈ *S*^⋆^, i.e., *D*_ε_ = ∅. Two subspaces *p*, *q* ∈ *S*^⋆^ are said to be **consistent** iff *p*(*v*) = *q*(*v*) for all *v* ∈ *D_p_* ∩ *D_q_*. We define the **intersection** *z*: = *q* ⊓ *p* of two consistent *p*, *q* ∈ *S*^⋆^ to be the unique *z* ∈ *S*^⋆^ that satisfies *S*[*z*] = *S*[*p*] ∩ *S*[*q*].

A **trap space** is a subspace that is also a trap set. Trap spaces are therefore trap sets with a particularly simple geometry. They generalize the notion of steadiness from states to subspaces. In Klarner et al. (2014), we proved that trap spaces are independent of the update strategy. It is therefore meaningful to denote the trap spaces of (*S*, ↪) by $S_{F}^{⋆}$ independent of →. If a network (*V*, *F*) satisfies $S_{F}^{⋆}=\left\{\varepsilon \right\}$, then we say it is **trap-space-free**. We also showed that the dynamics inside a trap space *p* is fully specified by the **reduced network** (*V_p_*, *F_p_*) with

$$V_{p}:=\left\{v\in V|v\notin D_{p}\right\},\;F_{p}:=\left\{f_{i}\left[p\right]|f_{i}\in F:v_{i}\notin D_{p}\right\}$$

where *f* [*p*] denotes the Boolean expression that is obtained by substituting the values *p*(*v*) for *v* ∈ *D_p_* into *f*  ∈ *F*, as introduced in Section 2.1 of Klarner et al. (2014).

Since every trap set contains at least one attractor, inclusion-wise minimal trap spaces can be used to predict the location of a particular attractor. Hence, we define a partial order on *S*^⋆^ based on whether the referenced subspaces are nested: *p* ≤ *q* iff *S*[*p*] ⊆ *S*[*q*]. The **minimal** trap spaces are defined by $\min\left(S_{F}^{⋆}\right):=\left\{p\in S_{F}^{⋆}|∄\;q\in S_{F}^{⋆}:\;q<p\right\}$.

### 2.3 CTL model checking

Model checking is a formal method from computer science to determine whether a transition system satisfies a temporal specification. See Carrillo et al. (2012) for a review of its application to computational biology.

A **transition system** is a 5-tuple *TS* = (*S*, →, *AP, L, I*) where (*S*, →) is a state transition graph, *AP* a set of atomic propositions, *L* : *S* → 2*^AP^* a labeling function and *I* ⊆ *S* a set of initial states. We use the atomic propositions *AP* : = {*v* = *c*, δ*_v_* = *d* | v ∈ *V, c* ∈ B, *d* ∈ {−1, 0, 1}} and define the labeling function *L* by

$$\begin{array}{ll} v=c\in L\left(x\right) & :\Leftrightarrow x\left(v\right)=c\\ \delta_{v}=d\in L\left(x\right) & :\Leftrightarrow f_{v}\left(x\right)-x\left(v\right)=d \end{array}$$

for all *c* ∈ B, *d* ∈ {−1, 0, 1} and *x* ∈ *S*. The label δ*_v_* = *d* therefore indicates whether a variable *v* is decreasing, steady or increasing in a state. In addition to “ =” we need the inequality operator “≠”, e.g., *v* ≠ *c* ∈ *L*(*x*) iff *x*(*v*) ≠ *c*, and the special atom *true* which satisfies *true* ∈ *L*(*x*) for all *x* ∈ S.

Next, we define a fragment of the temporal specification language CTL that is sufficient for the subsequent sections. A formula φ of this fragment is defined by

$$\varphi ::=a\left|\right.\varphi_{1}\wedge \varphi_{2}\left|\right.\varphi_{1}\vee \varphi_{2}\left|\right.\text{EF}\left(\varphi \right)\left|\right.\text{AG}\left(\varphi \right)$$

where α ∈ *AP*, **EF** is the “exists finally” operator and **AG** the “always globally” operator. The semantics of the operators and the satisfaction relation ⊧ for transitions systems and CTL formulas is defined in Table 1. Since the atomic propositions and labeling function are fixed for the remainder of this article, we will specify transition systems by 3-tuples *TS* = (*S*, →, *I*). In practice, we use the model checking tool nusmv (Cimatti et al., 2000) to decide whether a given transition system satisfies a CTL query.

**Table 1:** **The satisfaction relation ⊧ for CTL formulas φ, states *x* ∈ *S*, and transition systems *TS* = (*S*, → , *AP, L, I* )**.

*x* ⊧ *a*	: ⇔	*a* ∈ *L*(*x*)
*x* ⊧ *φ*_1_∧*φ*_2_	: ⇔	*x* ⊧ *φ*_1_ and *x* ⊧ *φ*_2_
*x* ⊧ *φ*_1_∨*φ*_2_	: ⇔	*x* ⊧ *φ*_1_ or *x* ⊧ *φ*_2_
*x* ⊧ **EF**(*φ)*	: ⇔	∃π ∈ *InfPaths*(*x*) : ∃*i* ∈ N_0_ : π[*i* ] ⊧ *φ*
*x* ⊧ **AG**(*φ)*	: ⇔	∀π ∈ *InfPaths*(*x*) : ∀*i* ∈ N_0_ : π[*i* ] ⊧ *φ*
*TS* ⊧ *φ*	: ⇔	∀*x* ∈*I*: *x* ⊧ *φ*

## 3 The Attractor Detection Problem

The naive approach to find all attractors of a given network, i.e., a full exploration of its STG, is limited by the state explosion problem. Several groups have developed tools and algorithms that address this problem. They may be grouped into those for deterministic updates (Irons, 2006; Dubrova and Teslenko, 2011; Akutsu et al., 2012; Veliz-Cuba et al., 2014) and non-deterministic updates (Garg et al., 2008; Skodawessely and Klemm, 2011; Berntenis and Ebeling, 2013). The average running times are usually given in terms of randomly generated networks and a connectivity parameter *k* that describes the distribution of in-degrees in the interaction graph. It seems that finding deterministic attractors is easier than non-deterministic attractors. Intuitively, computing the terminal SCCs of digraphs with all out-degrees equal to one is easier than for digraphs with higher out-degrees. The average running times for synchronous STGs with hundreds of variables is, for example, on the order of seconds with the tool bns (Dubrova and Teslenko, 2011), which is based on a variant of *bounded linear time logic* (LTL) model checking and uses a *satisfiability* (SAT) solver to detect attractors.

Algorithms for non-deterministic STGs, on the other hand, are likely to run for hours or days for networks with less than even 100 variables (see Section 2). Garg et al. (2008) and the tool genysis is based on the symbolic manipulation of reachable states using *binary decision diagrams* (BDDs), while Skodawessely and Klemm (2011) and Berntenis and Ebeling (2013) rely on a guided exploration and enumeration of the state space.

### 3.1 Attractor detection pre-process

If *v* ∈ *V* is a constant with *f_v_* = *c* and *A* an attractor, then *x* (*v*) = *c* for every *x ∈ A*. Hence, before we start an attractor detection algorithm, we may safely remove all constants. The result is a reduced network whose attractors are in a one-to-one relationship with the attractors of the original network. During the removal of constants, update functions that depend on them may in turn become constant. The pre-process is therefore improved by an iterative substitution until there are no more constants.

The **percolation** operator $\vec{•}:S_{F}^{⋆}\to S_{F}^{⋆}$ is defined on the set of trap spaces by the following recursion. Let *p* be the initial trap space, for example, defined by the constants *C* ⊆* V* of a network (*D_p_* : = *C* and *p*(*v*) : = *f_v_*). The initial percolation is ${\vec{p}}_{0}:=p$ and for each *k* ∈ N_0_ we define ${\vec{p}}_{k+1}$ by

$$\begin{array}{ll} D_{{\vec{p}}_{k+1}} & :=\left\{v\in V|f_{v}\left[{\vec{p}}_{k}\right]\;\text{is}\;\text{constant}\right\}\\ {\vec{p}}_{k+1}\left(v\right) & :=f_{v}\left[{\vec{p}}_{k}\right],\;\text{for}\;\text{all}\;v\in D_{{\vec{p}}_{k+1}}. \end{array}$$

Note that *f* [*p*] denotes the Boolean expression obtained by substituting the values *p*(*v*) into *f*, as introduced in Section 2.1 of Klarner et al. (2014). Because ${\vec{p}}_{0}=p$ it follows that ${\vec{p}}_{k+1}\leq {\vec{p}}_{k}$ and ${\vec{p}}_{k}\in S_{F}^{⋆}$, for all *k* ∈ N_0_. Since *V* is finite, there is some *K* ∈ N_0_ such that ${\vec{p}}_{K}={\vec{p}}_{K+1}$ and $\vec{p}:={\vec{p}}_{K}$ is well-defined. Percolations are cheap to compute and have the following implication for the location of attractors (see Siebert (2011)):

**Proposition 1**. If *p* is a trap space and *A* ⊆ *S*[*p*] an attractor of (*S*, ↪), then $A\subseteq S\left[\vec{p}\right]$.

In the following sections, we will assume that the initial network is constant-free.

### 3.2 Attractor detection by random walks

Given a trap space *p*, for example, the whole space *p* = ε, we can find an attractor *A* ⊆ *S*[*p*] by a sufficiently long random walk (*x*_0_, *x*_1_, … , *x*_k_) where *x*_0_ ∈ *S*[*p*]. In practice, we use *k* = 10|*V* | and found that so far, without exception, random paths of this length have reached an attractor. To decide whether *x_k_* does really belong to an attractor we use the CTL query of 2. It uses the CTL formula *φ_p_* defined by $\varphi_{p}:=\wedge_{v\in D_{p}}\left(v=p\left(v\right)\right)$ if *p* ≠ ε, and *φ_p_* = *ture* otherwise.

**Proposition 2** (Attractor State). Let *p* be a trap space and *x* ∈ *S*[*p*]. The state *x* belongs to an attractor *A* ⊆ *S*[*p*] of (*S*, ↪) iff

$$\mathrm{TS}=\left(S_{V_{p}},↪,\left\{y\right\}\right)⊧\text{AG}\left(\text{EF}\left(\varphi_{y}\right)\right)$$

where $y\in S_{V_{p}}$ is the projection of *x* ∈ *S_V_* onto *V_p_*, i.e., *y*(*v*) : = *x*(*v*) for all *v* ∈ *V_p_*.

Starting from *x* ∈ *A*, we can then enumerate *A* by listing all states reachable from *x*. Note that model checking is performed on the reduced system $\left(S_{V_{p}},↪\right)$ rather than the full system (*S*, ↪) and that there is no equivalent LTL query to decide whether *x* belongs to an attractor (**G**(**F**(*φ_y_*)) does not work). Also, the observation that finding a single attractor is easy using a random walk does not contradict the fact that finding all attractors is hard.

## 4 Approximating Attractors by Subspaces

The result of attractor detection algorithms are usually sets of states that make up each attractor. The notion of an approximation of an attractor is instead based on information regarding steady and oscillating variables. An **approximation** of the attractors of a STG is a set *P* ⊆ *S*^⋆^ such that each *S*[*p*] contains an attractor. The trivial approximation for any network is *P* : = {ε}. Approximations differ in what can be learned from them about the number of attractors and their locations. The best approximation for a single attractor is the smallest subspace it is contained in. The **smallest subspace** that contains *A* ⊆ *S* is *p* ∈ *S*^⋆^ defined by *D_p_* : = {*v* ∈ *V*  | ∀*x*, *y* ∈ *A* : *x*(*v*) = *y*(*v*)} and *p*(*v*) : = *x*(*v*) for *x* ∈ *A* arbitrary. We denote it by *Sub*(*A*). Note that in general, *A* ≠ *Sub*(*A*) and that there may be two attractors *A*, *B* ∈ *S* with *A* ≠ *B* such that *Sub*(*A*) = *Sub*(*B*). The quality of an approximation is defined in terms of the following criteria.

**Definition 1**. A subspace *p* is **faithful** in (*S*, ↪) iff *Sub*(*A*) = *p* for every attractor *A* ⊆ *S*[*p*] of (*S*, ↪). An approximation *P* is faithful iff each *p* ∈ *P* is faithful.

**Definition 2**. A subspace *p* is **univocal** in (*S*, ↪) iff there is a unique attractor *A* of (*S*, ↪) such that *A* ⊆ *S*[*p*]. An approximation *P* is univocal iff each *p* ∈ *P* is univocal.

**Definition 3**. An approximation *P* is **complete** in (*S*, ↪) iff for every attractor *A* ⊆ *S* of (*S*, ↪) there is *p* ∈ *P* such that *A* ⊆ *S*[*p*].

Note that the three properties are independent of each other. If *P* is faithful, univocal, and complete, then we call it a **perfect approximation**. If *P* is perfect, then all attractors can be found by the random walk method above.

**Figure 1:**
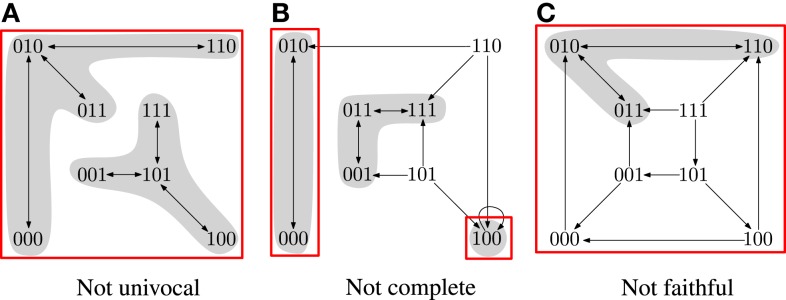
**The asynchronous STGs of three different Boolean networks**. The minimal trap spaces are indicated by boxes. **(A)** Two attractors in the same box. **(B)** An attractor outside of the boxes. **(C)** An attractor that does not oscillate in all dimensions of the box. Equations for the networks are given in the Supplementary Material.

In Klarner et al. (2014), we observed that $\min\left(S_{F}^{⋆}\right)$ is a good candidate for a perfect approximation. We showed that steady states are minimal trap spaces ($S_{F}\subseteq \min\left(S_{F}^{⋆}\right)$) and that every $p\in \min\left(S_{F}^{⋆}\right)\setminus S_{F}$ contains only cyclic attractors. Given that $\min\left(S_{F}^{⋆}\right)$ can be computed efficiently using ASP, we would like to have an efficient method for determining its quality as an approximation. Figure 1 demonstrates that $\min\left(S_{F}^{⋆}\right)$ is, in general, neither univocal, complete nor faithful.

### 4.1 Univocality

**Proposition 3** (Univocality). Let *p* be a trap space and *x* ∈ *A* such that *A* ⊆ *S*[*p*] is an attractor of (*S*, ↪). *p* is univocal in (*S*, ↪) iff

$$\mathrm{TS}=\left(S_{V_{p}},↪,S_{V_{p}}\right)⊧\text{EF}\left(\varphi_{y}\right)$$

where $y\in S_{V_{p}}$ is the projection of *x* ∈ *S_v_* onto *V_p_*.

The intuition behind this proposition is that if *A* is the only attractor inside the trap space *p* then *x* must be reachable from all states $S_{V_{p}}$.

### 4.2 Faithfulness

**Proposition 4** (Faithfulness). A trap space *p* is faithful in (*S*, ↪) iff

$$\mathrm{TS}=\left(S_{V_{p}},↪,S_{V_{p}}\right)⊧\underset{v\in V_{p}}{\wedge }\text{EF}\left(\delta_{v}\neq 0\right).$$

This proposition is true because a variable *v* oscillates in an attractor *A* iff there is a state *x* ∈ *A* such that *x* ⊧ (*δ_v_* ≠ 0).

### 4.3 Completeness

**Proposition 5** (Completeness). A set of trap spaces *p* is complete in (*S*, ↪) iff

$$\mathrm{TS}=\left(S,↪,S\right)⊧\underset{p\in P}{\vee }\text{EF}\left(\varphi_{p}\right).$$

Although we may restrict the initial states to *S* ∖ ∪*_p ∈ P_* *S*[*p*], the completeness query is still essentially dealing with the whole transition system and is therefore much less efficient than the queries of Proposition 2–4 (which are decided on reduced systems). In Klarner (2015b), we benchmarked nusmv and found that Boolean networks with *n* ≈ 39–55 variables may be considered infeasible for queries of this type. The next section develops a refinement-based approach to decide completeness that can deal with much larger networks.

## 5 Deciding Completeness by Iterative Refinement

The central idea for the refinement-based approach is to exploit hierarchies in the interaction graph and to use model checking on subnetworks that are in the upper layers of the hierarchy rather than the whole network. Given a complete set of trap spaces *p*, we keep replacing each *p* ∈ *P* by smaller trap spaces until either $P=\min\left(S_{F}^{⋆}\right)$ and we declare victory, or we find some *p* ∈ *P* that satisfies the failure criterion below which implies that $\min\left(S_{F}^{⋆}\right)$ can not be complete.

**Proposition 6** (Refinement). Let $P\subseteq S_{F}^{⋆}$ be complete in (*S*, ↪) and *p* ∈ *P* some trap space. If $Q\subseteq S_{F_{p}}^{⋆}$ is complete in $\left(S_{V_{p}},↪\right)$ then P′:=(P∖{p})∪{p⊓q|q∈Q} is complete in (*S*, ↪).

Note that the intersection *p* ⊓ *q* is necessary to position the trap space *q* of $\left(S_{V_{p}},↪\right)$ correctly in the full transition system (*S_V_*, ↪) and that (*p* ⊓ *q*) ≤ *p*. An example of a refinement is the percolation operator. By Proposition 1, if *P* is complete, then $\vec{P}:=\left\{\vec{p}|p\in P\right\}$ is also complete. The **failure criterion** is based on the observation that if $\min\left(S_{F}^{⋆}\right)$ is complete in $\left(S_{V_{p}},↪\right)$, then $\min\left(S_{F_{p}}^{⋆}\right)$ must be complete in $\left(S_{V_{p}},↪\right)$ for every $p\in S_{F}^{⋆}$.

**Proposition 7** (Failure Criterion). If there is a trap space *p* such that $\min\left(S_{F_{p}}^{⋆}\right)$ is not complete in $\left(S_{V_{p}},↪\right)$, then $\min\left(S_{F}^{⋆}\right)$ is not complete in (*S*, ↪).

**Example 1**. Consider the network defined by *V* = {*v*_1_, *v*_2_, *v*_3_} and *F* with $f_{1}=\bar{v_{1}}\bar{v_{2}}+v_{1}v_{2}+v_{2}\bar{v_{3}}$, $f_{2}=\bar{v_{1}}\bar{v_{2}}v_{3}+v_{1}v_{2}v_{3}$ and *f*_3_ = *v*_2_ + *v*_3_. The minimal trap spaces are {111,^⋆^00}. The trap space *p* : = ^⋆⋆^1 satisfies the failure criterion because $\min\left(S_{F}^{⋆}\right)=\left\{11\right\}$ is not complete in $\left(S_{V_{p}},↪\right)$ as there is, for example, no path from 01 to 11 in $\left(S_{V_{p}},↪\right)$. It follows that $\min\left(S_{F}^{⋆}\right)$ is not complete.

### 5.1 Autonomous sets

To find the initial $P\subseteq S_{F}^{⋆}$ and then $Q\subseteq \min\left(S_{F_{|U}}^{⋆}\right)$ for a given *p* ∈ *P* we use Proposition 8 below. It is based on so-called autonomous sets, a generalization of inputs. The variables *U* ⊆ *V* are **autonomous** iff *Above*(*U*) = *U* in the interaction graph. An autonomous *U* induces a **restricted network** (*U*, *F_|U_*) where *F*_|_*_U_* : = { *f*_u_ ∈ *F* | *u* ∈ *U*}. Note that if *U* is autonomous, then (*U*, *F*_|_*_U_*) is a well-defined network.

**Proposition 8**. Let *U* be autonomous and $Q:=\min\left(S_{F_{|U}}^{⋆}\right)$ the minimal trap spaces of the restriction (*U*, *F_|U_*).

(a) If *Q* is complete in (*S_U_*, ↪), then *Q* is also complete in (*S*, ↪).
(b) If *Q* is not complete in (*S_U_*, ↪), then $\min\left(S_{F}^{⋆}\right)$ is not complete in (*S*, ↪).

Note that the inputs *I* ⊆*V* of a network are autonomous and that *P* defined by $P:=\left\{p\in S_{F}^{⋆}|D_{p}=I\right\}$ (the |*P*| = 2^|^*^I^*^|^ input combinations) is complete in (*I*, *F*_|_*_I_*). Proposition 8(a) implies that *P* is also complete in (*V*, *F*). $\vec{P}$ is a refinement of *P* and if any $\vec{p}\in \vec{P}$ satisfies the failure criterion then $\min\left(S_{F}^{⋆}\right)$ is not complete.

**Example 2**. Consider the network with *V* = {*v*_1_, … ,*v*_4_} and *F* with *f*_1_ = *v*_1_, *f_2_* = *v_2_*, $f_{3}=v_{1}\bar{v_{4}}$, *f*_4_ = *v*_2_*v*_3_. The minimal trap spaces are {0000, 0100, 1000, 11^⋆⋆^}. To decide whether they are complete we observe that the network has two inputs {*v*_1_, *v*_2_} and four input combinations whose minimal trap spaces are *P* = {00^⋆⋆^, 01^⋆⋆^, 10^⋆⋆^; 11^⋆⋆^}. Since $\vec{P}=\min\left(S_{F}^{⋆}\right)=\left\{0000,0100,1000,11^{⋆⋆}\right\}$, we deduce that $\min\left(S_{F}^{⋆}\right)$ is complete.

### 5.2 Minimal autonomous sets

A refinement-based algorithm requires choosing an autonomous set *U* and deciding whether *Q* is complete in (*S_U_*, ↪) using the query of Proposition 5. The best performance in terms of model checking is expected if the minimal sets are as small as possible. **Minimal autonomous sets** (set-inclusion-wise) are located in the top layer of the interaction graph (*V*, →) and can be found using any SCC algorithm.

**Proposition 9**. Let *U*⊆*V*. The following statements are equivalent:

(a) *U* is a minimal autonomous set of (*V*, →).
(b) *U* is autonomous and *U* ∈ *SCCs*(*V*, →).

Once it is confirmed that the minimal trap spaces of each restriction are complete, we may consider their **intersection**.

**Proposition 10**. If $P,Q\subseteq S_{F}^{⋆}$ are complete in (*S*, ↪) then P⊓Q:={p⊓q|p∈P, *q* ∈ *Q*: *p* and *q* are consistent} is also complete in (*S*, ↪).

Note that if *P* and *Q* are complete, then for each *p* ∈ *P*, there is necessarily a *q* ∈ *Q* such that *p* and *q* are consistent. Similarly, for each attractor *A* ⊆ *S*[*p*], there is some consistent *q* ∈ *Q* such that *A* ⊆ *p* ⊓ *q*. Hence *P* ⊓ *Q* is non-empty and complete. Also, unless there is *p* ∈ *P* with *p* ∈ *Q* we get |*P* ⊓ *Q*| = |*P*| ⋅ |*Q*|. Finally, inputs are minimal autonomous sets and if a network has no other minimal autonomous sets, then the intersection is equal to the input combinations. Taking the intersection therefore generalizes the approach of inputs and input combinations.

**Example 3**. Consider the network with *V* = {*v*_1_, … ,*v*_6_} and *F* with *f*_1_ = *v*_2_, *f*_2_ = *v*_1_, *f*_3_ = *v*_4_, *f*_4_ = *v*_3_, $f_{5}=v_{2}\bar{v_{6}}$ and *f*_6_ = *v*_3_*v*_5_. The minimal trap spaces are $\min\left(S_{F}^{⋆}\right)=\left\{000000,001100,110010,1111^{⋆⋆}\right\}$. The network has two minimal autonomous sets *U*_1_ = {*v*_1_, *v*_2_} and *U*_2_ = {*v*_3_, *v*_4_}. The corresponding restrictions are $\left(U_{1},F_{|U_{1}}\right)$ and $\left(U_{2},F_{|U_{2}}\right)$ with the minimal trap spaces $Q_{1}:=\min\left(S_{F_{|U}}^{⋆}\right)=\left\{11,00\right\}$ and $Q_{2}:=\min\left(S_{F_{|U}}^{⋆}\right)=\left\{11,00\right\}$. Model checking (or inspection of the STGs) confirms that they are complete in their respective restricted systems. The intersection $P:=Q_{1}⊓Q_{2}$ and the percolation $\vec{P}$ are $P=\left\{0000^{⋆⋆},0011^{⋆⋆},1100^{⋆⋆},1111^{⋆⋆}\right\}$ and $\vec{P}=\left\{000000,001100,110000,1111^{⋆⋆}\right\}$. As before in Example 2, $\vec{P}=\min\left(S_{F}^{⋆}\right)$ and we deduce that $\min\left(S_{F}^{⋆}\right)$ must be complete in (*S*, ↪).

### 5.3 Extending minimal autonomous sets

Although minimal autonomous sets are favorable for efficient model checking, there is no guarantee that the respective restricted systems do actually contain non-trivial trap spaces. A refinement based on the trivial trap space ε, i.e., *Q* = {ε}, is useless because it means replacing *p* with *p* ⊓ ε = *p*, that is, with itself. A possible solution is to increase the size of checked autonomous sets until we find non-trivial trap spaces. The question is: by how many variables should we extend an autonomous set *U*? On the one hand, we want to be generous because new variables increase the chances for finding new trap spaces. On the other hand, we want to add as few variables as possible because the failure criterion requires CTL model checking.

What is the best extension for a given *U* whose restricted system is trap-space-free? Adding only outputs or cascades to *U* is not enough as the emergence of trap spaces requires “self-freezing”, positive feedback circuits, see Section 4.7 in Klarner (2015b). Intuitively, we want to extend down to the next SCC.

For a clean definition, we introduce the following notions. The set of **cascade components** consists of all single element SCCs in the interaction graph, whose nodes do not have self-loops. The remaining components are the **non-cascade components**.

$$\begin{array}{ll} \mathrm{Casc}\left(V,\to \right) & :=\left\{U\in \mathrm{SCCs}\left(V,\to \right)|\exists v\in V:U=\left\{v\right\},v↛v\right\}\\ \mathrm{NonCasc}\left(V,\to \right) & :=\mathrm{SCCs}\left(V,\to \right)\setminus \mathrm{Casc}\left(V,\to \right) \end{array}$$

The **condensation graph** (*Z*, ▹) of the interaction graph is then the digraph with vertices *Z* : = *NonCasc*(*V*, →) such that an arc *U* ▹ *W* indicates whether there is a cascade from *U* to *W*. More precisely, *U* ▹ *W* iff *U ≠ W* and there is *u* ∈ *U*, *w* ∈ *W* such that

∃π∈FinPaths(u,w):∀1≤i≤len(π)−2:{π[i]}∈Casc(V,→).

Note that (*Z*, ▹) is acyclic and so we can partition its vertex set into classes, which we call **layers**, depending on the longest path that reaches them.

Lay(W):=max{len(π)|π∈FinPaths(U,W),U∈Z}

Note that *Lay*(*W*) ≥ 1 because π = (*W*) is an admissible path from *W* to *W* and *len*(*W*) = 1 and that all minimal autonomous sets can then be found in the first layer of the condensation graph, i.e., *U* ⊆*V* is minimal and autonomous iff *U* ∈ *Z* and *Lay*(*U*) = 1.

**Figure 2:**
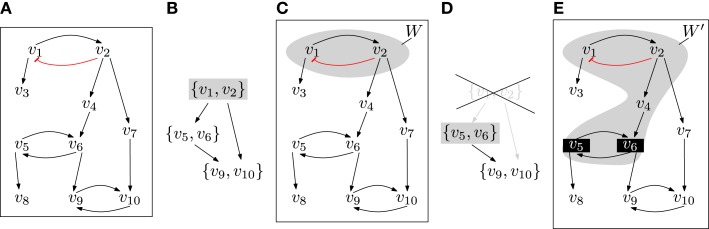
**(A)** The interaction graph of an example network where each *f_i_* is the disjunction of its inputs (e.g., *f*_6_ = *v*_5_ + *v*_4_) except for *v*_1_ which is inhibited by *v*_2_ ( $f_{1}:=\bar{v_{2}}$). **(B)** The condensation graph (*Z*, ▹) with the unique top layer node {*v*_1_ *v*_2_}. **(C)** The corresponding minimal autonomous set *W*. The restriction (*W*, *F*_|*W*_) is trap-space-free. **(D)** To extend *W*, we compute the graph (*Z*′, ▹), which is obtained from (*Z*, ▹) by removing the node that intersects *W*. The new top layer node is *Y*  : = {*v*_5_, *v*_6_}. **(E)** The extended autonomous set *W* ′ is obtained by considering *Above*(*Y*) in the interaction graph. It has a minimal trap space *p* that is defined by fixing *v*_5_ and *v*_6_ to 1.

To illustrate how the condensation graph is used for extending autonomous sets, consider the network given in Figure 2. First, we compute its minimal autonomous sets, i.e., the top layer of (*Z*, ▹). In this example, there is a unique *W*  ∈ *Z* with *Lay*(*W*) = 1. The restriction (W, F_|W_) consists of an isolated negative feedback circuit and is trap-space-free. To determine the smallest extension that contains new feedback circuits, we first compute the graph (*Z*′, ▹), which is obtained from the condensation graph (*Z*, ▹) by removing all *U* ∈ *Z* that satisfy *U* ∩ *W*  ≠ ∅. For each *Y*  ∈ *Z*′ that satisfies *Lay*(*Y*) = 1, we get an extended autonomous set *W* ′ by considering the variables above *Y* in the interaction graph (*V*, →). In the example, there is again a unique *Y* and the restriction to *W* ′ : = *Above*(*Y*) contains a non-trivial trap space *p*. The failure criterion is not satisfied by *p* and so we have found an initial complete set, namely *P* : = {*p*}. Note that in general, there will be several minimal autonomous sets and several possible extensions. We are now ready to design an efficient algorithm for deciding completeness.

### 5.4 The algorithm

**Figure 3:**
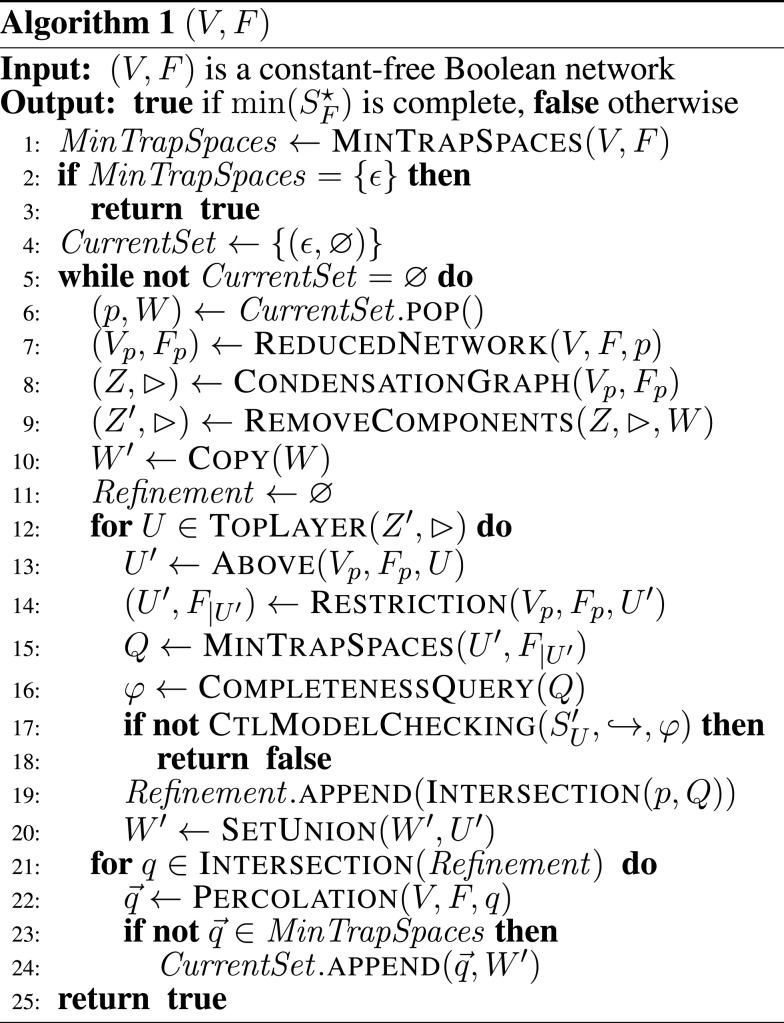
**The iterative, refinement-based algorithm for deciding the question of completeness**. See main text for a detailed description.

The first step of the algorithm in Figure 3 is to compute the minimal trap spaces of a given network using the ASP-based method proposed in Klarner et al. (2014). If the network is trap-space-free, then $\min\left(S_{F}^{⋆}\right)=\left\{\varepsilon \right\}$ is, by definition, complete and we stop and return *true*. Otherwise the variable *CurrentSet* is initialized. It consists of tuples (*p*, *W*), where *p* is a trap space and *W*  ⊆ *V* are the variables of the network (*V_p_*, *F_p_*) that have previously been subjected to model checking. The tuples correspond to those trap spaces of a complete set that need further refinement (i.e., are not minimal). Initially *CurrentSet* : = {(ε, ∅)} because {ε} is trivially complete and we have not started model checking *W*  = ∅. The lines 5–24 execute the iterative refinement of *CurrentSet* until we either find a *p* that satisfies the failure criterion in line 17 or *CurrentSet* = ∅ in which case every *p* is equal to some minimal trap space (only non-minimal trap spaces are put back onto *CurrentSet*, see lines 23, 24).

The next steps are to select an arbitrary (*p*, *W*) for refinement (line 6), compute the reduced network (*V_p_*, *F*_p_), its condensation graph (*Z*, ▹) and the graph (*Z*′, ▹) described in the previous section. The top layer elements *U* of (*Z*′, ▹) are minimal autonomous sets if *Z* = *Z*′ or extended autonomous sets if *Z* ≠ *Z*′. In the latter case, the restricted networks that correspond to minimal autonomous sets of (*V_p_*, *F_p_*) must have previously been found to be trap-space-free. For each *U*, the variables above *U* are autonomous (in (*V_p_*, *F_p_*)). If the minimal trap spaces of the restricted networks are complete in ($S_{U^{\prime }},↪$) then, by Proposition 8(a), they are also complete in ($S_{V_{p}}$, ↪). Otherwise it follows, by Proposition 8(b), that $\min\left(S_{F_{p}}^{⋆}\right)$ is not complete in ($S_{V_{p}}$, ↪) and hence that *p* satisfies the failure criterion and we stop and return *false* in line 18.

The variable *Refinement* stores all complete sets that were found in the upper layers of (*V_p_*, *F_p_*), while *W* ′ keeps track of the variables that were subjected to model checking. Line 21 is an application of Proposition 10, i.e., the intersection of all complete sets is taken (generalization of input combinations). For each trap space *q* in the intersection, we check whether the percolation $\vec{q}$ needs further refinement (not a minimal trap space of (*V*, *F*)) and if so add it back onto *CurrentSet*.

Note that (*V_p_*, *F*_p_) must have non-trivial trap spaces for each (*p*, *W*) ∈ *CompleteSets* (see lines 23, 24). Hence, although it may happen that (*p*, *W*) is replaced by (*p*, *W* ′) (if *Q* = {ε} in line 15) eventually it will be replaced by smaller trap spaces. The algorithm is implemented and available as part of our python toolbox for Boolean networks (Klarner, 2015a).

### 5.5 Counterexamples for attractor detection

If $\min\left(S_{F}^{⋆}\right)$ is not a perfect approximation, we would like to know why. Model checking tools like nusmv are capable of producing a counterexample in case a formula does not hold. Intuitively, a counterexample is a finite path from an initial state that proves that the query is false. If $\min\left(S_{F}^{⋆}\right)$ is not complete, then the algorithm of the previous section can be used to return some $p\in S_{F}^{⋆}$ that satisfies the failure criterion together with a counterexample to the respective completeness query for (*V_p_*, *F_p_*) and min($S_{F_{p}}^{*}$). Every attractor that is reachable from its last state, say *x*, must then be outside of min($S_{F_{p}}^{*}$). We then use the random walk approach to find state a *z* that belongs to an attractor *A* ⊆ *S*_*V*_*p*__ outside of min $\left(S_{F_{p}}^{*}\right)$. If the modified completeness query

$$\mathrm{TS}=\left(S_{V_{p}},↪,S_{V_{p}}\right)⊨\varphi_{z}\vee \underset{q\in \min\left(S_{F}^{⋆}\right)}{\vee }\text{EF}\left(\varphi_{q}\right)$$

holds then *A* is the only outside attractor, otherwise we use the next counterexample to find the next outside attractor until they are all found. Note that *p* is an extension of a minimal autonomous set. A similar approach is possible for trap spaces that are not faithful or not univocal. We end up with a set of states that captures the attractors outside of *P*, the number of attractors inside *S*[*p*] for each *p* ∈ *P* and whether they are faithful or not.

## 6 Results

All computations in this section were done on a 32-bit Linux laptop with 4 × 2.60 GHz and 8 GB memory.

### 6.1 MAPK case study

In this case study, we consider the network published in Grieco et al. (2013), which models the influence of the MAPK pathway on cancer cell fate decisions and consists of 53 variables. Using Klarner (2015a), we compute $\min\left(S_{F}^{⋆}\right)$ in under one second. It consists of 12 steady states and six trap spaces that contain only cyclic attractors. The single query approach to deciding completeness runs 35 min, while the refinement-based algorithm confirms completeness in only 28 s. For the six trap spaces in $\min\left(S_{F}^{⋆}\right)$ ∖ *S_F_* we confirmed univocality in 261 s (44 s on average per trap space) and faithfulness in 74 s (12 s on average per trap space) using the CTL queries of Section 4. Hence, $\min\left(S_{F}^{⋆}\right)$ is a perfect approximation of the attractors of (*S*, ↪) and for each attractor we can find an internal state by the random walk approach of Section 4. We stopped genysis after seven hours without a result.

**Figure 4:**
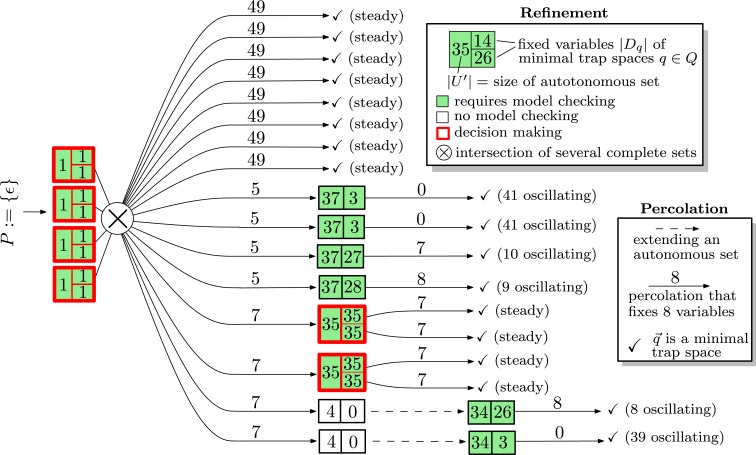
**An illustration of the how the iterative refinement algorithm confirms that $\min\left(S_{F}^{⋆}\right)$ of the MAPK network is complete**. Altogether 12 restricted systems are model checked instead of the single full system. A more detailed description is given in the main text.

Figure 4 is an illustration of the steps performed during the iterative refinement for the MAPK network. The information is represented as a **decision tree**. The root represents the initial and trivially complete set *P* : = {ε}. Boxes are split into a left side, representing the size |*U*| of a minimal autonomous set (or an extension), and a right hand side that is split vertically into cells that contain the numbers |*D_q_*| of fixed variables for each minimal trap space *q* of (*U*, *F*_|_*_U_*). Boxes are colored according to whether (*U*, *F*_|_*_U_*) is trap-space-free (white) or not in which case model checking is required to find out whether the minimal trap spaces of (*U*, *F*_|_*_U_*) are complete (failure criterion). Boxes with more than one minimal trap space are outlined in red to emphasize that a decision process between competing trap spaces exists. The intersection of several autonomous sets is indicated by ⊗ but occurs for this network only for the inputs. Arcs are labeled by the number of variables that are fixed during percolations, i.e., $\left|D_{q}\setminus D_{\vec{q}}\right|$ (see line 22 in Figure 3). If a restricted network is trap-space-free, the extension is indicated by a dashed arc. Along each branch of the decision tree, the number of fixed and oscillating variables must add up to 53. The bottom branch, for example, starts with four fixed variables, percolates seven more, extends an autonomous set whose restriction consists of four variables and is trap-space-free, finds a single trap space with three fixed variables and finishes as the remaining trap space is minimal (and 4 + 7 + 3 + 0 + 39 = 53).

Note that the algorithm encounters roughly four types of refinements. The first type (branches 1–8) leads directly to a steady state. The second type (branches 9–12) discovers a single minimal autonomous set consisting of 37 variables, whose restriction has a single minimal trap spaces in which between nine and 41 variables oscillate. The third type (branches 13–14) discovers a single minimal autonomous set that has two minimal trap spaces that commit the network to different steady states. The fourth type (branches 15–16) discovers a single minimal autonomous set consisting of four variables that is trap-space-free. An extension leads an autonomous set of 34 variables with a single minimal trap space.

### 6.2 GINsim repository benchmark

To test whether the MAPK network is unusual in that its minimal trap spaces are perfect approximations, we ran the same analysis for every Boolean model currently in the ginsim model repository (see Naldi et al. (2009)). In every case, the minimal trap spaces are perfect approximations of the attractors of (*S*, ↪). The time needed to confirm faithfulness, univocality and completeness is given in Table 2. We confirmed the number of steady states and cyclic attractors with ginsim and genysis. The execution of ginsim is, like the computation of minimal trap spaces, instantaneous. The running times of genysis are comparable to that of our algorithm for networks with |V| < 40 and on the order of 24–72 h for the three networks with |*V* | ≥ 40. The networks and attractors are available for benchmarking at Klarner (2015a).

**Table 2:** **The minimal trap spaces of all Boolean models in the ginsim repository are perfect approximations of the attractors of (*S*, ↪)**.

Network file (.zginml)	|*V* |	Steady	Cyclic	Faithful	Univocal	Complete
buddingYeastOrlando2008	9	1	–	0.08*s*	0.03*s*	0.23*s*
fissionYeastDavidich2008	10	12	–	0.01*s*	0.02*s*	0.08*s*
boolean_cell_cycle	10	1	1	0.03*s*	0.19*s*	0.12*s*
Toll_Pathway_12Jun2013	11	4	–	0.01*s*	0.01*s*	0.09*s*
drosophilaCellCycleVariants	14	1	–	0.01*s*	0.05*s*	0.11*s*
MAPK_red3_19062013	16	12	6	0.15*s*	1.11*s*	0.93*s*
MAPK_red1_19062013	17	12	6	0.18*s*	1.25*s*	0.87*s*
VEGF_Pathway_12Jun2013_0	18	256	–	0.04*s*	0.05*s*	0.28*s*
MAPK_red2_19062013	18	12	6	0.14*s*	1.26*s*	0.67*s*
buddingYeastIrons2009	18	–	1	0.16*s*	0.48*s*	0.02*s*
ErbB2_model	20	1	–	0.08*s*	0.00*s*	0.02*s*
FGF_Pathway_12Jun2013	23	512	–	0.09*s*	0.09*s*	0.51*s*
Hh---Pathway_11Jun2013_0	24	8192	–	1.29*s*	1.43*s*	6.34*s*
Spz---Processing_12Jun2013	24	64	–	0.04*s*	0.03*s*	0.22*s*
Wg_Pathway_11Jun2013	26	16384	–	2.38*s*	2.38*s*	17.16*s*
TCRsig40	40	7	1	1.07*s*	3.34*s*	0.12*s*
MAPK_large_19june2013	53	12	6	40.15*s*	565.84*s*	20.72*s*
T_LGL	60	86	70	1.07*s*	6.57*s*	5669.57*s*

*The number of variables, steady states, and cyclic attractors are recorded in the first three columns. The remaining three columns record the time needed to confirm faithfulness, univocality, and completeness*.

## 7 Conclusion and Outlook

In this paper, we developed the notion of an approximation of attractors of a Boolean network. Minimal trap spaces are approximations that can be computed for networks with hundreds of variables using ASP solvers. Since available attractor detection tools for asynchronous systems are only feasible for about 50 variables, approximations via minimal trap spaces might yield attractor information otherwise inaccessible. We defined three criteria to assess the quality of an approximation and showed that they can be decided using model checking. The main contribution in this paper is an algorithm that improves the efficiency of deciding completeness by dividing the problem into smaller subproblems according to autonomous sets in the interaction graph.

We ran the algorithm on the 18 Boolean networks that are currently in the ginsim repository and found that each time, the minimal trap spaces are a perfect approximation of the asynchronous attractors, i.e., that we can find all asynchronous attractors using random walks and $\min\left(S_{F}^{⋆}\right)$.

Section 5.3 explains that autonomous sets must be extended if the corresponding restricted systems are trap-space-free. Strategies by which extensions are constructed must compromise between adding variables to increase the likelihood of discovering non-trivial trap spaces and the efficiency of model checking the respective transition systems. The strategy in Section 5.3 can be considered optimal in the sense that it adds as few variables at a time as necessary for the emergence of new trap spaces.

There are several directions in which the algorithm may be improved further, for example, by removing so-called “mediator variables” (see, e.g., Saadatpour et al. (2013)) from the interaction graph of the subnetworks. The relationship to other reduction methods, e.g., Naldi et al. (2011) or Veliz-Cuba (2011), may also yield improvements by reducing the size of the transition systems passed to the model checking software further.

The decision tree in Figure 4 might be an interesting tool for questions regarding network control, an idea that was recently developed in Zañudo and Albert (2015). It also suggests that the dynamics of Boolean networks is governed by two very different regimes: the *percolation regime* in which the long-term activities are pre-determined, and the *decision-making regime* in which the long-term activities are determined by which of the competing trap spaces is reached first.

## Conflict of Interest Statement

The authors declare that the research was conducted in the absence of any commercial or financial relationships that could be construed as a potential conflict of interest.

## Supplementary Material

The Supplementary Material for this article can be found online at http://journal.frontiersin.org/article/10.3389/fbioe.2015.00130
