# Supplementary material for: Approximating Attractors of Boolean Networks by Iterative CTL Model Checking
Source: Front Bioeng Biotechnol. 2015 Sep 8;3:130. doi: 10.3389/fbioe.2015.00130 (PMC4562258; doi:10.3389/fbioe.2015.00130)
Supplement: Supplementary file 1 [file data_sheet_1.pdf]

Supplementary material for
"Approximating attractors of Boolean networks by iterative CTL model checking"

H. Klarner and H. Siebert
FB Mathematik und Informatik, Freie Universität Berlin
Arnimallee 6, 14195 Berlin, Germany

July 2015

1 Proofs for the propositions

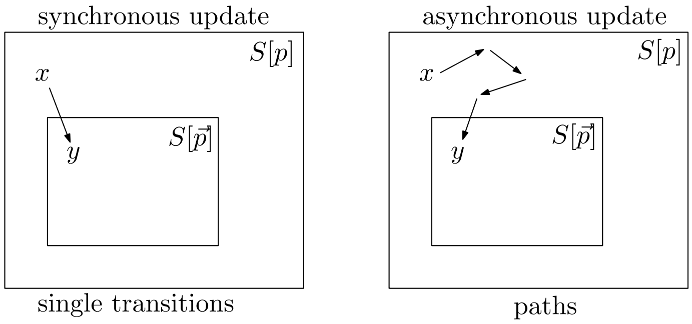

Figure 1: Since there every $x \in S[p]$ there is $y \in S[\vec{p}]$ such that there is a path from x to y there can not be an attractor that intersect $S[p] \setminus S[\vec{p}]$.

Proposition 1 (Fig. 1)**.** *If p is a trap space and $A \subseteq S[p]$ an attractor of $(S, \to)$ then $A \subseteq S[\vec{p}]$.*

Proof. The percolation $\vec{p}$ of a trap space p is defined by iterative substitution (see Sec. 3.1 in main text), i.e., by a sequence of trap spaces

$$p = p_0, p_1, p_2 \dots, p_K = \vec{p}$$

where each pair p_k, p_{k+1} is a single percolation step and K the first index that satisfies $p_K = p_{K+1}$. Witout loss of generality we can assume that $K = 1$ because the statement is trivially true for $K = 0$ and will follow for $K > 1$ by induction. Hence, let p be a trap space whose percolation is achieved by a single step.

Synchronous update: Any state in the subspace p will reach the subspace $\vec{p}$ by a single transition because $f_v(x) = \vec{p}(v)$ holds for any v in the domain of

$\vec{p}$ (by definition of $\vec{p}$). Since $\vec{p}$ is a trap space this implies that there can not be a SCC in between p and $\vec{p}$, i.e., intersecting $S[p] \setminus S[\vec{p}]$.

Asynchronous update: For any state x in the subspace p and any variable v that is fixed in $\vec{p}$ there is a transition to some state y such that $\vec{p}(v) = y(v)$. Since this argument can be repeated for y there is a path from x to the subspace $\vec{p}$ (of at most $|D_{\vec{p}} \setminus D_p|$ transitions). As before, since $\vec{p}$ is a trap space there can be no attractor in between p and $\vec{p}$. □

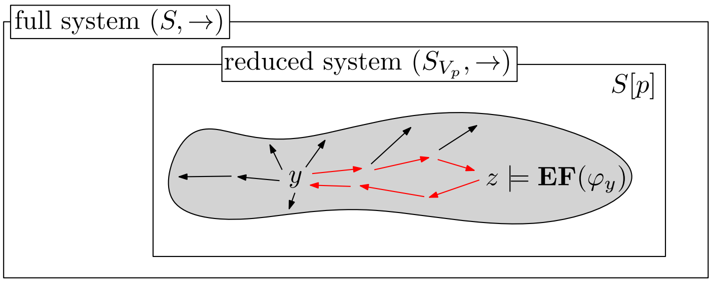

Figure 2: Iff all states z that are reachable from y satisfy $\mathbf{EF}(\varphi_y)$ then y belongs to an attractor $A \subseteq S[p]$.

Proposition 2 (Attractor State, Fig. 2)**.** *Let p be a trap space and $x \in S[p]$. The state x belongs to an attractor $A \subseteq S[p]$ of $(S, \to)$ iff*

$$TS = (S_{V_p}, \to, \{y\}) \models \mathbf{AG}(\mathbf{EF}(\varphi_y))$$

where $y \in S_{V_p}$ is the projection of $x \in S_V$ onto V_p, i.e., $y(v) := x(v)$ for all $v \in V_p$.

Proof. Let p be a trap space and $x \in S[p]$ with $x \in A$ for some attractor $A \subseteq [p]$. Since A is an attractor it is an inclusion-wise minimal trap set (by definition) and must therefore be strongly connected because otherwise it would contain a smaller trap set. Hence any state in A, and therefore any state reachable from x, has a path back to x. With respect to the reduced system $(S_{V_p}, \to)$ this means that any state $z \in S_{V_p}$ that is reachable from the projection y satisfies $\mathbf{EF}(\varphi_y)$. Since the states reachable from y are referenced by $\mathbf{AG}$ it follows that $\mathbf{AG}(\mathbf{EF}(\varphi_y))$ is true for y. So the transition system $(S_{V_p}, \hookrightarrow)$ with initial states $\{y\}$ satisfies $\models \mathbf{AG}(\mathbf{EF}(\varphi_y))$.

Let p be a trap space such that $TS = (S_{V_p}, \hookrightarrow, \{y\}) \models \mathbf{AG}(\mathbf{EF}(\varphi_y))$ where $y \in S_{V_p}$ is the projection of some state $x \in S_V$ onto V_p. Then all states reachable from y ($\mathbf{AG}$) have a path back to y ($\mathbf{EF}(\varphi_y)$ and hence y belongs to a strongly connected component A' (all states of A' are connected via y). A' must also be a trap set because the connectedness holds for every state reachable from y. Hence A' is an attractor. Note that $A' \subseteq S_{V_p}$ so far, but given p we can position A' in S_V, call it A, by assigning values to the variables D_p according to p such that $A \subseteq S_V$ is an attractor of $(S, \to)$ and $x \in A$. □

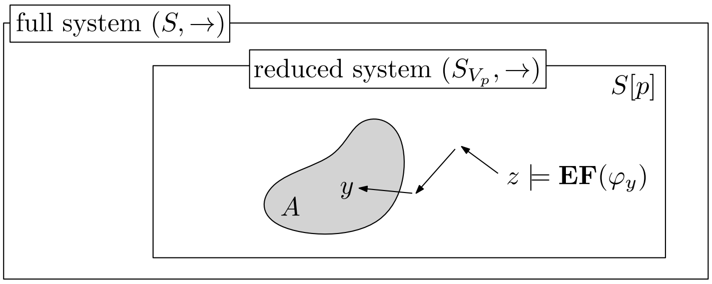

Figure 3: Iff for every state $z \in S_{V_p}$ there is a path to y then A is be the unique attractor of $S[p]$.

Proposition 3 (Univocality, Fig. 3)**.** *Let p be a trap space and $x \in A$ such that $A \subseteq S[p]$ is an attractor of $(S, \to)$. p is univocal in $(S, \to)$ iff*

$$TS = (S_{V_p}, \to, S_{V_p}) \models \mathbf{EF}(\varphi_y)$$

where $y \in S_{V_p}$ is the projection of $x \in S_V$ onto V_p.

Proof. If p is univocal in $(S, \to)$ then A is the only attractor of $(S, \to)$ and $x \in A$ can be reached from every state in S_{V_p}. Hence the transition system $(S_{V_p}, \to)$ with initial states S_{V_p} satisfies $\mathbf{EF}(\varphi_y)$.

If the transition system $TS = (S_{V_p}, \to, S_{V_p})$ satisfies $\mathbf{EF}(\varphi_y)$ then y belongs to the unique attractor $A' \subseteq S_{V_p}$ of $(S_{V_p}, \to)$. As in the previous proof we can use p to position A' in the original transition system $(S, \to)$ and this set A will be the unique attractor $A \subseteq S[p]$ and $x \in A$ holds. □

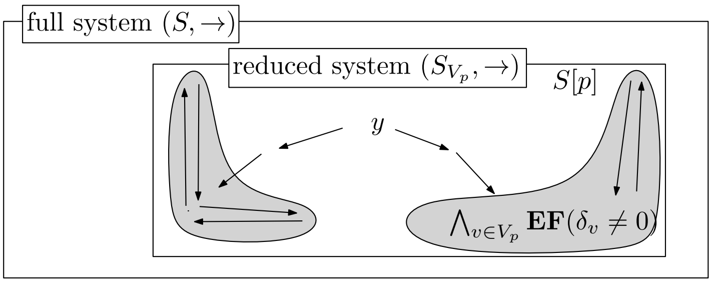

Figure 4: The attractors of a trap space p are faithful iff for every $y \in S_{V_p}$ and $v \in V_p$ there is a path to a state z that satisfies $z \models \delta_v \neq 0$.

Proposition 4 (Faithfulness, Fig. 4)**.** *A trap space p is faithful in $(S, \to)$ iff*

$$TS = (S_{V_p}, \to, S_{V_p}) \models \bigwedge_{v \in V_p} \mathbf{EF}(\delta_v \neq 0).$$

Proof. Let p be faithful and $x \in S_{V_p}$ arbitrary. We want to prove that

$$x \models \bigwedge_{v \in V_p} \mathbf{EF}(\delta_v \neq 0). \tag{1}$$

Since p is faithful, every attractor $A \subseteq S[p]$ satisfies $Sub(A) = p$. Let A be an attractor of $S[p]$ that is reachable from x. Since $Sub(A) = p$ there are $x_1, x_2 \in A$ such that $x_1(v) \neq x_2(v)$ for every $v \in V_p$. Since x_1, x_2 belong to A there is a path between x_1 and x_2 and hence a transition in which the activity of $v \in V_p$ changes. Let $x'_1 \to x'_2$ be such that $x'_1(v) \neq x'_2(v)$. Hence $\delta_v(x'_1) \neq 0$. Since A is reachable from x and x'_1 from x it follows that $x \models \mathbf{EF}(\delta_v \neq 0)$. Since $v \in V_p$ was chosen arbitrarily, Eq. 1 holds.

For the other direction let the transition system $(S_{V_p}, \to)$ with initial states S_{V_p} be such that Eq. 1 holds for every $x \in S_{V_p}$. The equation therefore holds in particular for every $x \in A$ where A is an attractor of $S[p]$. Hence, for every $v \in V_p$ and attractor A there is $y \in A$ such that $\delta_v(y) \neq 0$ and hence a transition $y \to y'$ such that $y(v) \neq y'(v)$. Hence $Sub(A) = p$ and so p is faithful. $\square$

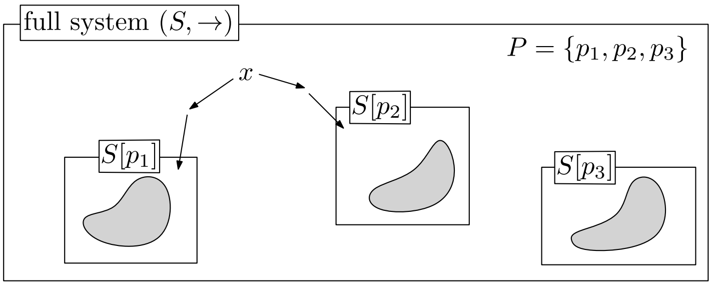

Figure 5: The trap spaces $P := \{p_1, p_2, p_3\}$ are complete iff for every initial state x there is a path to some trap space $p \in P$.

Proposition 5 (Completeness, Fig. 5)**.** *A set of trap spaces P is complete in $(S, \to)$ iff*

$$TS = (S, \to, S) \models \bigvee_{p \in P} \mathbf{EF}(\varphi_p).$$

Proof. Let P be a complete set or trap spaces of $(S, \to)$ and $x \in S$ arbitrary. We want to show that

$$x \models \bigvee_{p \in P} \mathbf{EF}(\varphi_p). \quad (2)$$

Let A be an arbitrary attractor that is reachable from x. Since P is complete there is $p \in P$ such that $A \subseteq S[p]$. Since there is a path from x to A it follows that $x \models \mathbf{EF}(\varphi_p)$ and therefore Eq. 2 holds.

For the other direction note that if Eq. 2 holds for all $x \in S$ that it holds in particular for all states of every attractor. But if for every attractor A there is a $p \in P$ such that there is a path from A to $S[p]$ then $A \subseteq S[p]$ and P is complete. $\square$

Proposition 6 (Refinement of Complete Sets, Fig. 6)**.** *Let $P \subseteq S^\star_F$ be complete in $(S, \to)$ and $p \in P$ some trap space. If $Q \subseteq S^\star_{F_p}$ is complete in $(S_{V_p}, \to)$ then $P' := (P \setminus \{p\}) \cup \{q \sqcap p \mid q \in Q\}$ is complete in $(S, \to)$.*

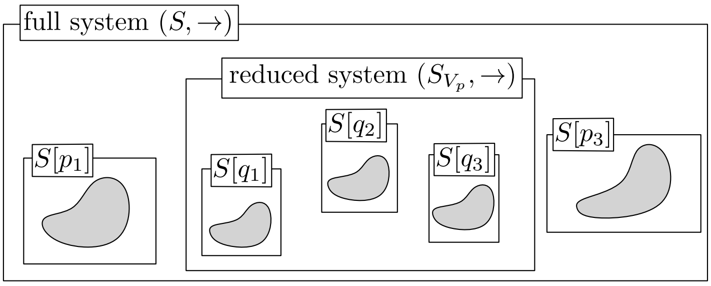

Figure 6: Refinement works by replacing a trap space p by some set of trap spaces that is complete in S_{V_p}.

Proof. Let P be a complete set of trap spaces of $(S, \rightarrow)$ and $p \in P$ arbitrary. Consider the reduced system (F_p, V_p) and its trap spaces $S^\star_{F_p}$ and let $Q \subseteq S^\star_{F_p}$ be complete in $(S_{V_p}, \rightarrow)$. Note that we defined subspaces as mappings $p : D_p \rightarrow \mathbb{B}$. Hence, although a trap space q of (V_p, F_p) is well-defined when considered as a subspace of (V, F), we need to intersect it with p to assign values to the variables that are implicitly fixed in q when considered as a subspace of (V_p, F_p). The completeness of P' then follows from the completeness of Q in $(S_{V_p}, \rightarrow)$ because the dynamics inside p is identical with the dynamics of the reduced system (V_p, F_p). □

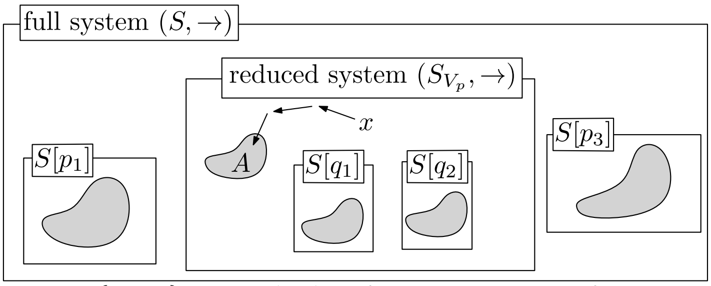

Figure 7: .

Proposition 7 (Failure Criterion, Fig. 7)**.** *If there is a trap space p such that $\min(S^\star_{F_p})$ is not complete in $(S_{V_p}, \rightarrow)$ then $\min(S^\star_F)$ is not complete in $(S, \rightarrow)$.*

Proof. Suppose p is such that $Q := \min(S^\star_{F_p})$ is not complete in $(S_{V_p}, \rightarrow)$. The main observation is that $P := \{p \sqcap q \mid q \in \min(S^\star_{F_p})\} \subseteq \min(S^\star_F)$. That is, if the subspace Q are positioned correctly within $(S, \rightarrow)$, i.e., intersected with p, then they are also minimal trap spaces of (V_p, F_p). The statement then follows because if Q is not complete in $(S_{V_p}, \rightarrow)$ then there is a state $x \in S[p]$ that can

not reach any trap space in P. But, since p is a trap space x must reach some attractor A which is therefore outside of P and hence outside of $\min(S_F^\star)$ which implies that $\min(S_F^\star)$ is not complete in $(S, \to)$. $\square$

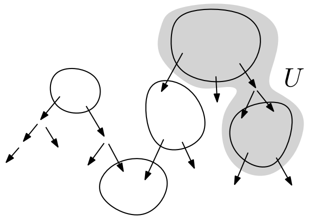

Figure 8: A schematic drawing of the interaction graph, enclosed are SCCs, and an autonomous set U.

Proposition 8 (Fig. 8)**.** *Let U be autonomous and $Q := \min(S^\star_{F_{|U}})$ the minimal trap spaces of the restriction $(U, F_{|U})$.*

(a) If Q is complete in $(S_U, \to)$ then Q is also complete in $(S, \to)$.
(b) If Q is not complete in $(S_U, \to)$ then $\min(S_F^\star)$ is not complete in $(S, \to)$.

Proof. **Observations:** The dynamics in the restricted and full transition systems can be related to each other. For any path $(y_0, y_1, \ldots, y_k)$ of $(S_U, \to)$ and any $x_0 \in S[y_0]$ there is a path $(x_0, x_1, \ldots, x_k)$ of $(S, \to)$ such that $x_i(u) = y_i(u)$ for all $u \in U$ and $1 \leq i \leq k$. Also, for any path $(x_0, x_1, \ldots, x_k)$ of $(S, \to)$ there is a unique path $(y_0, y_1, \ldots, y_r)$ in $(S_U, \to)$ with $r \leq k$, $x_0 \in S[y_0]$ and $x_k \in S[y_r]$ that describes the projected dynamics. It follows that a trap space q of $(U, F_{|U})$ is also a trap space of (V, F) because otherwise we could consider the projection of the path that proves that q is not a trap space in (V, F) and deduce that q is not a trap space in $(U, F_{|U})$, a contradiction. Hence Q is a set of trap spaces of (V, F).

Proof of (a): Let Q be complete in $(S_U, \to)$ and $x \in S$ an arbitrary state. We want to show that there is a path from x to some $q \in Q$. Let y be the projection of x onto U. Since Q is complete there is a path $(y_0, y_1, \ldots, y_k)$ such that $y_0 = y$ and $y_k \in S[q]$ for some $q \in Q$. By the observations above there is therefore a path $(x_0, x_1, \ldots, x_k)$ with $x_0 = x$ and $x_k \in S[q]$. Hence Q is complete in $(S, \to)$.

Proof of (b): The main observation is that since U is autonomous and since $Q = \min(S^\star_{F_{|U}})$ it follows that for any $p \in \min(S_F^\star)$ there is $q \in Q$ such that $p \leq q$. If Q is not complete in $(S_U, \to)$ then there is $y \in S_U$ that can not reach any $q \in Q$. Any x whose projection on U is equal to y can therefore not reach any $q \in Q$ in $(S, \to)$. Hence it can not reach any $p \in \min(S_F^\star)$ (because for any p there is a $q \in Q$ with $q \leq p$). Hence $\min(S_F^\star)$ is not complete in $(S, \to)$. $\square$

Proposition 9 (Fig. 8)**.** *Let $U \subseteq V$. The following statements are equivalent:*

(a) U is a minimal autonomous set of $(V, \to)$.
(b) U is autonomous and $U \in SCCs(V, \to)$.

Proof. **(a) ⇒ (b):** Let U be minimal and autonomous in $(V, \rightarrow)$. We need to show that U is strongly connected. Let $u, v \in U$ be arbitrary. If there is no path from u to v then u is not above v and so $Above(v)$ is a proper autonomous subset U, a contradiction to minimality. Hence, there is a path from u to v and so U is stronlgy connected.

(b) ⇒ (a): Let U be autonomous and strongly connected. We need to show that U does not contain a smaller autonomous set. Assume there is $U' \subset U$ with $U' \neq U$ and U' is autonomous. Let $u \in U \setminus U'$. Since U is strongly connected there is a path from u to any $u' \in U'$. Hence u is above u' and so $u \in U'$ which contradicts $u \in U \setminus U'$. Hence such U' does not exist and U is minimal. □

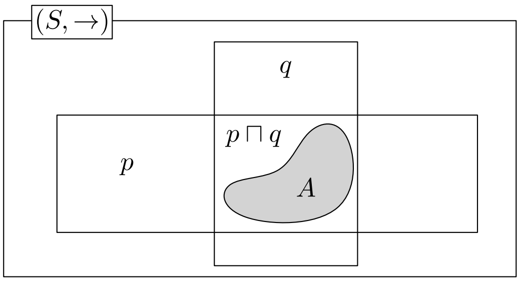

Figure 9: For every attractor A there are $p \in P$ and $q \in Q$ such that $A \subseteq S[p]$ and $A \subseteq S[q]$. Hence p and q are consistent and $A \subseteq S[p \sqcap q]$.

Proposition 10 (Fig. 9)**.** *If $P, Q \subseteq S^{\star}_{F}$ are complete in $(S, \rightarrow)$ then $P \sqcap Q := \{p \sqcap q \mid p \in P, q \in Q : p$ and q are consistent$\}$ is also complete in $(S, \rightarrow)$.*

Proof. Let A be an attractor of $(S, \rightarrow)$. Since P and Q are complete there are $p \in P$ and $q \in Q$ such that $A \subseteq S[p]$ and $A \subseteq S[q]$. Hence, p and q are consistent and $(p \sqcap q) \in P \sqcap Q$. Hence $P \sqcap Q$ is complete in $(S, \rightarrow)$. □

Proposition 11. *Let $(Z, \rhd)$ be the condensation graph of a constant-free network (V, F). A set $U \subseteq V$ is minimal and autonomous iff $U \in Z$ and $Lay(U) = 1$.*

Proof. Let U be minimal and autonomous. It follows from Prop. 9 that $U \in SCCs(V, \rightarrow)$. We need to show that $Lay(U) = 1$. If $Lay(U) > 1$ then $Above(U) \supseteq U$ with $Above(U) \neq U$ which contradicts U being autonomous.

For the other direction assume that $U \in Z$ and $Lay(U) = 1$. We will again use Prop. 9. Note that $Z = SCCs(V, \rightarrow)$. Also, U is autonomous because if $Above(U) \supset U$ with $U \neq Above(U)$ then $Lay(U) > 1$, i.e., there would have to be an SCC above U. Note that the last deduction uses the fact that (V, F) is constant-free. □

2 Update Functions

The update functions for the three Boolean networks are given in Fig. 10.

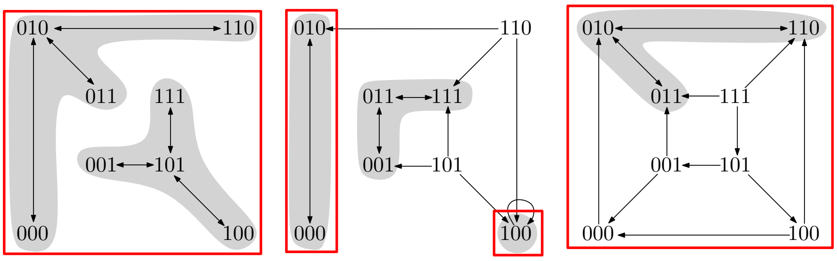

(a) Not univocal (b) Not complete (c) Not faithful

Figure 10: The asynchronous STGs of the three Boolean networks given in Fig. 1 of the main text.

$$\begin{aligned} f_1 &:= \overline{v_1}\,\overline{v_2}v_3 + \overline{v_1}v_2\overline{v_3} + v_1\overline{v_2}\,\overline{v_3} + v_1v_2v_3 \\ f_2 &:= \overline{v_1}\,\overline{v_2}\,\overline{v_3} + \overline{v_1}v_2v_3 + v_1\overline{v_2}v_3 + v_1v_2\overline{v_3} \\ f_3 &:= \overline{v_1}\,\overline{v_2}v_3 + \overline{v_1}v_2\overline{v_3} + v_1\overline{v_2}\,\overline{v_3} + v_1v_2v_3 \end{aligned} \tag{a}$$

$$\begin{aligned} f_1 &:= \overline{v_1}\,v_2v_3 + v_1\overline{v_2}\,\overline{v_3} \\ f_2 &:= \overline{v_1}\,\overline{v_2} + v_1v_3 \\ f_3 &:= \overline{v_1}\,v_3 + v_1v_2 \end{aligned} \tag{b}$$

$$\begin{aligned} f_1 &:= \overline{v_1}\,v_2\overline{v_3} \\ f_2 &:= \overline{v_1} + \overline{v_3} \\ f_3 &:= \overline{v_1}\,v_2\overline{v_3} \end{aligned} \tag{c}$$
